# Impact of Glucocorticoid Receptor Gene Polymorphisms on the Metabolic Profile of Adult Patients with the Classical Form of 21-Hydroxylase Deficiency

**DOI:** 10.1371/journal.pone.0044893

**Published:** 2012-09-18

**Authors:** Ricardo P. P. Moreira, Larissa G. Gomes, Berenice B. Mendonca, Tânia A. S. S. Bachega

**Affiliations:** Unidade de Endocrinologia do Desenvolvimento, Laboratório de Hormônios e Genética Molecular (LIM/42), Disciplina de Endocrinologia da Faculdade de Medicina da Universidade de São Paulo, São Paulo, Brazil; Ecole Normale Supérieure de Lyon, France

## Abstract

**Background:**

CAH patients have an increased risk of cardiovascular disease, and it remains unknown if lifelong glucocorticoid (GC) treatment is a contributing factor. In the general population, glucocorticoid receptor gene (*NR3C1*) polymorphisms are associated with an adverse metabolic profile. Our aim was to analyze the association between the *NR3C1* polymorphisms and the metabolic profile of CAH patients.

**Methodology:**

Sixty-eight adult patients (34SV/34SW) with a mean age of 28.4±9 years received dexamethasone (mean 0.27±0.11 mg/day) to obtain normal androgen levels. SW patients also received fludrocortisone (50 µg/day). Metabolic syndrome (MetS) was defined by the NCEP ATPIII criteria and obesity by BMI ≥30 kg/m^2^. *NR3C1* alleles were genotyped, and association analyses with phenotype were carried out with Chi-square, *t*-test and regression analysis.

**Results:**

Obesity and MetS were observed in 23.5% and 7.3% of patients, respectively, and were not correlated with GC doses and treatment duration. BMI was positively correlated with blood pressure (BP), triglycerides (TG), LDL-c levels and HOMA-IR and inversely correlated with HDL-c levels. *Bcl*I and A3669G variants were found in 26.4% and 9.6% of alleles, respectively. Heterozygotes for the *Bcl*I polymorphism presented with higher BMI (29 kg/m^2^±5.3 *vs.* 26 kg/m^2^±5.3, respectively) and waist circumference (89 cm±12.7 *vs.* 81 cm±13, respectively) compared to wild-type subjects. Hypertension was found in 12% of patients and heterozygotes for the *Bcl*I polymorphism presented higher systolic BP than wild type subjects. Low HDL-c and high TG levels were identified in 30% and 10% of patients, respectively, and were not associated with the *NR3C1* polymorphisms. A3669G carriers and non-carriers did not differ.

**Conclusion:**

In addition to GC therapy, the *Bcl*I GR variant might play an important role in obesity susceptibility in CAH patients. Genotyping of GR polymorphisms could result in the identification of a subgroup at risk patients, allowing for the establishment of personalized treatment and the avoidance of long-term adverse consequences.

## Introduction

Congenital adrenal hyperplasia (CAH) due to 21-hydroxylase deficiency is a common autosomal recessive disorder that leads to decreased glucocorticoid secretion, with or without mineralocorticoid deficiency, and to increased androgen production [1]. CAH is caused by mutations in the *CYP21A2* gene, which codes for 21-hydroxylase, a key enzyme involved in cortisol and aldosterone synthesis, and accounts for 90–95% of adrenal enzymatic defects [1], [2].

The spectrum of clinical manifestations depends on the degree of 21-hydroxylase impairment, including a severe form with prenatal virilization of the external genitalia in female fetuses and postnatal virilization in both sexes, with or without salt loss (classical forms), and a milder form with late onset hyperandrogenic signs (nonclassical). The classical form of CAH has a prevalence of about one in 15,000 to one in 16,000 live births in the general population [1], [2].

The introduction of glucocorticoid (GC) replacement therapy in the 1950s has allowed for a normal life span in CAH patients. Current GC therapy aims to provide adequate glucocorticoid replacement dose and to suppress the abnormal androgen secretion; mineralocorticoid replacement aims to control the renal salt balance to avoid adrenal crisis [1], [2], [3]. Nevertheless, these therapeutic goals are difficult to achieve in practice due to the complexity of replicating the physiologic circadian rhythm of cortisol secretion [4], [5].

Recent studies have demonstrated the increased prevalence of obesity, insulin resistance and hypertension [6], [7], [8], as well as adverse lipid profiles, among adult and pediatric CAH patients. These findings suggest that CAH patients are prone to developing an unfavorable cardiovascular risk profile [7]. CAH patients can also develop signs and symptoms of iatrogenic Cushing's syndrome [3], mainly due to the supraphysiological doses of GC used to suppress hyperandrogenism and chronically to reduce 17-hydroxyprogesterone (17-OHP) levels. Previous studies have associated the increased prevalence of obesity and hypertension with hypercortisolism [9]; however, other factors may be involved, such as individual glucocorticoid sensitivity.

Völkl *et al*. [10] demonstrated an important contribution of parental obesity to the increased body mass index (BMI) in children and adolescents with CAH, suggesting that genetic factors could be involved with obesity predisposition. On the other hand, none of the available studies have evaluated the role of genetic polymorphisms on the metabolic profiles of CAH patients.

In the general population, some glucocorticoid receptor (*NR3C1*) gene polymorphisms, are linked with increased BMI, blood pressure and lipid levels and, consequently, increased cardiovascular risk [11]. The *Bcl*I polymorphism, located at intron 2, has been associated with increased GC sensitivity and with these abovementioned clinical manifestations [11], [12], [13], [14], [15]. Another *NR3C1* polymorphism, the A3669G, is linked with increased expression and stabilization of the dominant negative splice variant GR-β, which results in relative GC resistance. Recent studies have suggested that A3669G carriers have an increased pro-inflammatory state and an increased risk for cardiovascular disease [11], [16].

Considering the variability in the prevalence of obesity and metabolic syndrome (MetS) in adult CAH patients, our aim was to evaluate whether *NR3C1* polymorphisms could account for the development of this adverse metabolic profile in a series of CAH patients from same center. We observed that *Bcl*I allele could influence the sensitivity to glucocorticoids in CAH patients, since their carriers presented higher BMI, waist circumference and systolic blood pressure in comparison with non-carriers.

## Methods

### Subjects

The study was approved by the Ethical Committee of Faculdade de Medicina da Universidade de São Paulo (0231/2010), and written consent was obtained from all the participants. The inclusion criteria were patients with the classical form of CAH, stable glucocorticoid and mineralocorticoid therapy in the last two years, no use of enzyme inductor drugs and good compliance, which was characterized by normal androgen and PRA levels in at least 3 out of 4 annual measurements.

From a cohort of 135 adult CAH patients, we selected 68 with a mean age of 28.4±8.6 years, who presented regularly at our endocrine service. Thirty-four patients (24 females) had the simple virilizing (SV) form, characterized by ambiguous genitalia in girls and postnatal virilization signs in both sexes. Thirty-four patients (24 females) had the salt wasting (SW) form, and they also presented with volume depletion, sodium levels <130 mmol/liter and increased plasma renin activity (PRA). All patients presented with a basal 17-OHP >150 nmol/L and molecular diagnosis of classical CAH [17].

After reaching their final height, all individuals were treated with dexamethasone (0.27±0.11 mg) once a day, doses of which are available in tablet (0.5 mg) and elixir (0.1 mg/mL) formulations. Mean daily glucocorticoid doses were calculated by body surface area (mg/m^2^) and evaluated retrospectively in the last 2 years. The glucocorticoid doses were converted to hydrocortisone equivalents using anti-inflammatory equivalents (30 mg hydrocortisone = 0.75 mg dexamethasone). The hydrocortisone equivalents are also presented as mg/m^2^. For the salt wasters, fludrocortisone was maintained at a mean dose of 50±25 mcg/day. The mean of duration of glucocorticoid therapy was 25.4±9.8 years.

### Hormonal Control

Treatment efficacy was assessed by hormonal control, characterized by normal androgen levels according to sex. The laboratory goals included the normalization of androstenedione and testosterone levels for females during the follicular phase of their menstrual cycle and androstenedione levels for men. The mineralocorticoid replacement was monitored by blood pressure and PRA, which was maintained in the upper half of the normal reference range [18].

PRA was measured using commercial kits (CIS-Bio International Gif-Sur-Yvette, France). Serum androstenedione levels were measured by chemiluminescence assay (Immulite 2000, Siemens Health Care, UK). Intra-assay and inter-assay coefficients of variation varied from 5% to 10%, respectively. Glucose was determined by an automatic enzymatic colorimetric method using hexokinase (Cobas Integra; Roche, Basel, Switzerland). Serum testosterone and insulin was determined by Auto Delfia fluoroimmunoassay (Perkin-Elmer, Turku, Finland). Total cholesterol (TC), high-density lipoprotein (HDL-c), low-density lipoprotein (LDL-c) and triglycerides (TG) levels were analyzed by an automatic enzymatic colorimetric method (Cobas Mira; F. Hoffmann-La Roche, Basel, Switzerland).

### Clinical, anthropometric and laboratory measurements

All patients underwent physical examination to obtain anthropometric measurements. Obesity was defined by a BMI ≥30 kg/m^2^ and overweight by a BMI between 25–29.9 kg/m^2^. Waist circumference was defined as abnormal if ≥102 cm in men and ≥88 cm in women.

Metabolic syndrome was defined according to the National Cholesterol Education Program, Adult Treatment Panel III criteria (NCEP ATPIII), with abnormal values for at least 3 of the 5 criteria: waist circumference, systolic or diastolic blood pressure, fasting glucose, HDL-c and triglycerides [19].

To evaluate the metabolic profile, blood samples were taken for the measurements of glucose, insulin, TC, HDL-c, LDL-c and TG at a fasting state, before the subjects took their hormonal replacement therapy.

Elevated blood pressure was defined as values ≥135/85 mmHg. Abnormal TG levels were characterized as values ≥150 mg/dL, HDL-c <40 mg/dL in men and <50 mg/dL in women and fasting glucose ≥110 mg/dL [19]. Insulin resistance was assessed by the homeostasis model assessment for insulin resistance (HOMA-IR).

### Genetic Analysis

DNA samples from the patients were obtained from peripheral blood leukocytes by salting out procedures. PCR amplification of the glucocorticoid receptor gene regions was carried out using primer sequences and amplification conditions as previously described [20], [21].

The A3669G polymorphism is located in the 3′ untranslated region of exon 9β, at nucleotide position 3669 (an A to G alteration), and was genotyped by sequencing. PCR products were sequenced using the Big Dye Terminator Sequencing Kit™ (Applied Biosystem, Inc., Foster City, CA, USA) and capillary electrophoresis on an ABI PRISM 3100 sequencer (Applied Biosystem, Inc.).

The ER22/23EK polymorphism comprises two linked, single nucleotide variations separated by one base pair in exon 2. The first substitution at nucleotide position 198 is silent, changing codon 22 from GAG to GAA. The second mutation changes codon 23 at nucleotide position 200 from AGG to AAG. The N363S polymorphism changes codon 363 of exon 2 at nucleotide position 1220 from AAT to AGT. Sequence traces were analyzed using Sequencher (version 4.5 build 1416).

The *Bcl*I polymorphism results in an intronic C to G change, 646 nucleotides downstream from exon 2. It was screened by an allele-specific PCR as previously described [21]. The results of the allele-specific PCR were confirmed by direct sequencing in 20 patients.

### Statistical analysis

Comparison of genotypes frequencies between different groups of patients and sex was carried out using a χ^2^ test. Normal distribution for all continuous variables was tested, and some were logarithmically transformed. At baseline, independent *t-*test for independent groups was applied to compare continuous variables. Results are reported as means ± SD. These analyses were also performed with adjustment for age and sex by multivariable modeling.

Pearson's correlation coefficients were used to calculate correlations between BMI, GC dose, insulin, HOMA, and lipid levels after correction for age and sex. Correlation analysis was also applied to compare the mean androgen levels of the least two years of GC therapy with BMI, waist circumference, BP and metabolic profile. *P*<0.05 was considered to indicate a significant difference.

Hardy–Weinberg equilibrium for the *Bcl*I and A3669G variants was calculated. Statistical analysis was performed using the software SigmaStat version 3.5 for Windows (Systat Software, Point Richmond, CA).

## Results

The clinical and anthropometric data of CAH patients are described in Table 1. Allelic frequencies of *Bcl*I and A3669G *NR3C1* polymorphisms were in Hardy-Weinberg equilibrium. The *Bcl*I polymorphism was found in 26.4% of the alleles, with 6 homozygous patients and 24 heterozygous patients. The A3669G polymorphism was found in 9.6% of the alleles, in 11 heterozygous patients and only 1 homozygous patient. The N363S and ER22/23EK alleles were identified in 1 patient as heterozygous for each polymorphism. For statistical analysis, we selected only polymorphisms with a frequency ≥5% (*Bcl*I and A3669G).

**Table 1:** Clinical and anthropometric characteristics of 68 adult CAH patients.

Variables	Female (N = 48)	Male (N = 20)	*P* value
Age, mean [SD], years	29.2 (9.5)	26.9 (6.8)	0.449
BMI, mean [SD], kg/m^2^	25.7 (4.1)	29.5 (7.1)	*0.035*
Increased WC, n (%)	7 (14.6)	7 (35)	0.238
GC dose, mean [SD], mg/m^2^	11.2 (4.5)	10.5 (3.7)	0.696
Duration of GC therapy, years	26.6 (10.6)	21.9 (7.2)	*0.039*
HOMA-IR index, mean [SD]	2.24 (1.02)	2.5 (1.7)	0.376
Total cholesterol, mean [SD], mg/dL	174 (35)	186.3 (33.3)	0.186
HDL-c, mean [SD], mg/dL	58.2 (13.2)	49 (12)	*0.010*
LDL-c, mean [SD], mg/dL	100.2 (28)	116.8 (26.9)	*0.033*
Triglycerides, mean [SD], mg/dL	78.4 (40.5)	102.7 (45.8)	*0.014*
Systolic blood pressure, mean [SD], mmHg	118.7 (18.9)	123.5 (8.3)	*0.042*
Diastolic blood pressure, mean [SD], mmHg	76.9 (6.7)	80.1 (6.8)	0.074

Values are given as means ± SD. BMI: Body Mass Index; GC, glucocorticoid; WC: Waist Circumference.

### Obesity and metabolic syndrome prevalence in adults with CAH

Obesity was observed in 16 CAH patients (23.5% of patients), being more prevalent in the males (n = 9, 56%), and in similar frequencies between the SW and SV groups (50%). Overweight was observed in 23 patients (33.8% of patients), and it was more frequent in the patients with the SW form (n = 13, 56.5%) and in females (n = 18, 78.3%). Metabolic syndrome was observed in 7.3% of patients; three out of 5 patients presented with the simple virilizing form, and 3 were male.

### Clinical and biochemical markers of metabolic risk

BMI was positively correlated with higher systolic and diastolic blood pressures, triglycerides, LDL-c levels and HOMA-IR values (*P*<0.01), and it was inversely correlated with HDL-c levels (*P* = 0.03). There were no correlations among BMI, GC dose, duration of therapy and androstenedione and testosterone levels, although the latter measurements were inversely correlated with HDL-c levels in female patients (testosterone r = −0.45, *P*<0.01; androstenedione r = −0.629, *P*<0.01). The remaining components of MetS were not correlated with androstenedione levels in both sexes.

As expected, the male patients presented with worse metabolic profiles compared to the females, characterized by increased WC values, higher BMI, systolic blood pressure, LDL-c and TG levels and lower HDL-c levels (Table 1). Increased TG levels (≥150 mg/dL) were observed in 10% of the patients (n = 7), with 4 out 7 patients presenting with the SV form. Low HDL-c levels were observed in 19 patients (30% of cases), with higher frequency in the SW form compared to the SV form (n = 12, 63%). Hypertension was identified in 8 patients (12%) and was more prevalent in the SV patients (n = 5, 71.4%). Increased waist circumference values were observed in 14 patients, being more prevalent in the SV patients (n = 9, 64%) and with similar frequencies between both sexes (n = 7 both, 50%).

**Figure 1:**
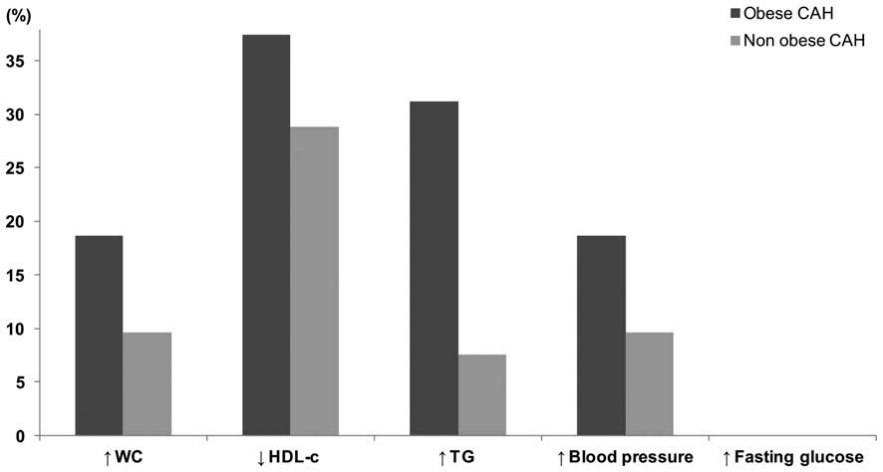
Comparison of frequency of metabolic syndrome components between CAH obese and CAH non-obese patients according to NCEP ATPIII criteria.

Decreased serum HDL-c levels were the most frequent component of the metabolic syndrome (30%) identified, followed by increased waist circumference (23.5%), high blood pressure (12%) and serum triglycerides levels (10%). Fasting plasma glucose level higher than 110 mg/dL was not observed in this series. The frequencies of the metabolic syndrome components, such as increased TG levels and blood pressure, were higher in the obese patients compared to the non-obese patients (Figure 1, *P*<0.05), independent of sex, age, GC dose, clinical form and duration of treatment.

### Impact of NR3C1 polymorphisms on the metabolic profile of CAH patients

Comparison of the clinical and laboratory data between carriers and non-carriers of the *Bcl*I polymorphism is shown in Table 2. *Bcl*I carriers presented with higher BMI, waist circumference and systolic blood pressure compared to wild-type carriers in the *t-test* analysis. These results were also adjusted according to sex and age in the linear multiple analyses and remained statistically significant (Table 2). We also found that *Bcl*I heterozygous carriers also presented higher BMI (29 kg/m^2^±5.3 *vs.* 23.9 kg/m^2^±2.7, *P* = 0.03), waist circumference (89 cm±12.7 *vs.* 72.8 cm±5.6, *P* = 0.005) and TG levels (91.3 mg/dL±45.5 *vs.* 50.7 mg/dL±27.8, *P* = 0.009) as compared to *Bcl*I homozygous carriers. There were no significant differences observed in the HOMA-IR value and lipid profile between carriers and non-carriers of the *Bcl*I polymorphism (Table 2).

**Table 2:** Impact of *Bcl*I polymorphism on the metabolic profile of CAH patients.

	Genotypes					
Variable	*Bcl*I (homozygous) n = 6	*Bcl*I (heterozygous) n = 24	Wild type n = 38	*P* value^a^^,^ b	Adjusted^b^ *P* value	Confidence^b^ Interval CI 95%
BMI, mean [SD], kg/m^2^	23.9 (2.7)	29 (5.3)	26 (5.3)	*0.018*	*0.044*	1.00–1.24
Waist circumference, mean [SD], cm	72.8 (5.6)	89 (12.7)	81 (13)	*0.011*	*0.044*	1.00–1.09
GC dose, mean [SD], mg/m^2^	11.2 (4.4)	9.9 (3.7)	11.6 (4.6)	0.137	0.164	0.78–1.04
Duration of GC therapy, mean [SD], y	22 (5.5)	25.6 (9.5)	25.4 (10.8)	0.240	0.461	0.91–1.21
HOMA-IR index	2.3 (1.5)	2.3 (1.4)	2.4 (1.2)	0.452	0.854	0.62–1.47
Serum total cholesterol, mean [SD], mg/dL	159.2 (27.2)	177 (39)	180.9 (32.8)	0.669	0.728	0.98–1.01
Serum HDL, mean [SD], mg/dL	63 (15.5)	52 (14.4)	56.5 (12.2)	0.111	0.216	0.93–1.02
Serum LDL, mean [SD], mg/dL	86 (19.6)	106.3 (30.6)	107.3 (27.8)	0.224	0.901	0.98–1.02
Serum triglycerides, mean [SD], mg/dL	50.7 (27.8)	91.3 (45.5)	87.4 (42.2)	*0.009*	0.828	0.99–1.01
Systolic blood pressure, mean [SD], mmHg	123 (6.7)	124.9 (7.1)	116.7 (8.9)	*<0.001*	*0.008*	1.03–1.29
Diastolic blood pressure, mean [SD], mmHg	75.3 (5.6)	80.1 (7.1)	76.8 (6.5)	0.064	0.179	0.97–1.16

Values are given as means ± SD.

Adjusted for age and sex.

a Univariated analysis.

b Comparison between *Bcl*I heterozygous and wild type carriers.

BMI: Body Mass Index; GC: glucocorticoid.

The frequency of the *Bcl*I polymorphism was higher in the obese patients compared to the non-obese patients (62.5% *vs.* 38.4%, respectively), and the frequency was also higher in the patients presenting with the metabolic syndrome (80% *vs.* 41.3%, respectively); however, these differences were not statistically significant.

Table 3 shows the clinical and laboratorial data of A3669G and wild-type carriers, and no differences were identified in cardiovascular risk factors such as BMI, waist circumference, blood pressure, HOMA-IR and lipid profile. There was no significant difference in the frequency of the A3669G polymorphism between the obese and non-obese CAH patients, 12.5% *vs.* 19.2%, respectively, or between the patients with and without metabolic syndrome, 20% *vs.* 17.4%, respectively.

**Table 3:** Impact of A3669G polymorphism on the metabolic profile of CAH patients.

	Genotype				
Variables	A3669G n = 12	Wild type n = 56	*P* value^a^	Adjusted *P* value	Confidence Interval - CI 95%
BMI, mean [SD], kg/m^2^	26.8 (5)	26.8 (5.5)	0.942	0.974	0.88–1.13
Waist circumference, mean [SD], cm	83.3 (12.2)	83 (13.5)	0.797	0.997	0.95–1.05
GC dose, mean [SD], mg/m^2^	10.6 (5)	11.1 (4.2)	0.727	0.947	0.84–1.18
Duration of GC therapy, mean [SD], y	22.8 (12.1)	25.6 (9.4)	0.631	0.621	0.85–1.05
HOMA-IR index, mean [SD]	2.1 (0.7)	2.4 (1.4)	0.427	0.594	0.47–1.54
Serum total cholesterol, mean [SD], mg/dL	192.7 (24.4)	174.4 (35.9)	0.097	0.192	0.99–1.04
Serum HDL-c, mean [SD], mg/dL	58.8 (9.5)	54.8 (14.1)	0.346	0.370	0.97–1.08
Serum LDL-c, mean [SD], mg/dL	115.6 (28.1)	102.8 (28.3)	0.161	0.219	0.99–1.04
Serum triglycerides, mean [SD], mg/dL	88.6 (35.1)	84.9 (45.1)	0.398	0.897	0.98–1.02
Systolic blood pressure, mean [SD], mmHg	121.5 (13.2)	119.8 (7.9)	0.600	0.718	0.93–1.11
Diastolic blood pressure, mean [SD], mmHg	78.5 (8)	77.7 (6.5)	0.708	0.974	0.89–1.11

Values are given as means ± SD.

Adjusted for age and sex.

a Univariated analysis.

## Discussion

The introduction of glucocorticoid therapy 60 years ago has allowed for a normal life span to CAH patients; however, increasing attention has been paid to the adverse long-term health effects of chronic hyperandrogenism and/or glucocorticoid therapy on the metabolic profile [7], [8], [22]. There is evidence of the increased prevalence of overweight/obesity in CAH patients compared to the normal population [6], [7], [23], and there is debate on whether long-term GC exposure or higher GC doses might be contributing factors.

In a multicenter study from the United Kingdom, a higher prevalence of obesity was observed in CAH patients (41% of patients) in comparison with the reference population. However, these patients received different regimes of glucocorticoids and suppressed 17-OHP levels (<12 nmol/L) were observed in 45% and 37% of CAH females and males, respectively [6]; thus, over-treatment could contribute to the development of obesity. Although in our series we selected patients followed in a same center and under a homogenous glucocorticoid replacement and hormonal control, we also found a higher frequency of obesity in comparison with our reference population (25), but it was lower than the former study (7). The aim of GC substitution therapy in our series was just to normalize the androgen levels, but not 17-OHP, and therefore the mean 17-OHP levels were higher (175.2 nmol/L) than the United Kingdom series and probably the mean daily GC doses could be lower in our patients. Considering these findings, we also speculated whether GC doses and/or duration of therapy differed between the obese and non-obese CAH patients and no significant differences were observed.

According to the MetS prevalence in our cohort, it was similar to the reference population from the same region [24]. Other CAH studies presented discordant results, and thus, we could not rule out an effect of different sample sizes, GC regimes and aims in the hormonal control as well as the use of different criteria in defining cardiovascular risk factors [6], [25], [26]. As it occurs in the general population, several factors could be involved with cardiovascular risk in CAH patients, such as obesity, hypertension and familial and/or genetic predisposition. Many works have analyzed the role of the *Bcl*I and A3669G *NR3C1* polymorphisms on the cardiovascular risk in the general population. The *Bcl*I polymorphism has been linked to increased GC sensitivity and consequently to higher BMI, waist circumference and lipid levels, compared to wild type carriers [11]; however, there are no data about the impact of these *NR3C1* polymorphisms on the metabolic profile of CAH patients.

In this study, we tested the association analysis between *NR3C1* polymorphisms and these traditional cardiovascular risk factors in patients treated exclusively with dexamethasone and similar hormonal control. Our results are consistent with previously published data from the general population, because our CAH patients carrying the *Bcl*I allele presented with increased BMI and waist circumference, independent of sex and age (Table 2). Additionally, the frequency of the *Bcl*I polymorphism was higher in the obese CAH compared to the non-obese CAH, but without statistical significance; thus, a sample size effect cannot be excluded.

The *Bcl*I allele has been also associated with higher blood pressure values and/or higher prevalence of hypertension [15] and similarly our CAH patients carrying this allele also presented higher systolic blood pressure levels. Probably, this result was not directly influenced by the dexamethasone therapy, considering that it has little mineralocorticoid effect [27]. Based on the data of our statistical analysis, the *Bcl*I allele and BMI were the major factors influencing the blood pressure values.

The fact that *Bcl*I heterozygous carriers presented with increased BMI, waist circumference and systolic blood pressure compared to two subgroups of homozygous carriers (mutant and wild-type) is in line with the observation in a previous study in which patients carrying the heterozygous *Bcl*I polymorphism gained more than twice the subcutaneous adiposity compared to homozygous subjects [28]. As hypothesized by Tremblay *et al*. [28], this phenomenon can be explained by a possible linkage disequilibrium between the *Bcl*I polymorphism and other polymorphisms located at regulatory regions that could influence the observed phenotype. Moreover, the compound heterozygosity could modify the GR responses to glucocorticoid, which was not observed in the homozygous carriers. This theory of the heterozygous phenotype is supported by two animal models, in which only the heterozygous and not the homozygous carriers develop the expected phenotype [29], [30]. However, it is important to emphasize that few patients were homozygous for this variant in our series and a sample size effect cannot be excluded.

Additionally, our results suggest that *Bcl*I CAH carriers might need lower GC doses (Table 2); but probably due to sample size, this result did not reach statistical significance. Supporting this hypothesis, it was observed that, due to increased GC sensitivity associated with the *Bcl*I polymorphism, patients with inflammatory bowel disease carrying this variant present a better response to glucocorticoid therapy [31].

The A3669G polymorphism is associated with increased expression and stabilization of the 9β GR isoform, and it is correlated with glucocorticoid resistance. A recent study suggested the involvement of this polymorphism with the increased risk of cardiovascular disease in carriers compared to non-carriers. This hypothesis was supported by the findings of elevated levels of inflammatory parameters, such as IL-6 and C reactive protein, in a large cohort of elderly subjects [16]. Our CAH patients carrying the A3669G polymorphism, although they present with similar mean BMI, total cholesterol, LDL-c and triglycerides levels were higher than compared to non-carriers. However, only 12 patients carried this allele and this finding was not statistically significant (Table 3).

Besides the genetic predisposition and glucocorticoid exposure, other factors could predispose to an adverse metabolic profile in CAH patients and as expected BMI presented significant influence on HOMA-IR, lipid levels and blood pressure, which were reinforced by the finding that obese CAH patients presented with higher frequency of MetS components than the non-obese patients. Another important factor is long-term hyperandrogenic exposure, which might be an independent contributor to the development of the metabolic syndrome's components. In a cohort of untreated female patients with the simple virilizing form was observed lower insulin sensitivity and higher body weight, blood pressure, and more metabolic disorders, including higher serum TG, and lower HDL-c [22] than the controls. To exclude the effects of increased androgens levels, we selected only patients with adequate hormonal control, and interestingly, mean androgen levels over the last 2 years of therapy were inversely correlated with lower HDL-c levels in our female patients. Although these patients presented normal androgen levels, glucocorticoid therapy probably does not reproduce or allow a normal adrenal androgen secretion.

The findings of our study suggest that the *Bcl*I polymorphism could play an important role in the susceptibility for obesity and higher systolic blood pressure in CAH patients and the positive correlation between *Bcl*I polymorphisms and BMI also suggests that the different sensitivities and individual responses to glucocorticoids are at least partially genetically determined [32]. Hence, GR screening during the treatment of CAH patients could help to improve the quality of GC replacement, by identifying subgroup patients at-risk who would benefit the most from personalized treatment. In these patients, we speculate that attempts to reduce 17-OHP levels significantly could predispose them to worse metabolic consequences, and we encourage the application of preventive measures.

## References

[1] Speiser PW, White PC (2003) Congenital adrenal hyperplasia. N Engl J Med 349: 776–788. 12930931 10.1056/NEJMra021561

[2] Merke DP, Bornstein SR (2005) Congenital adrenal hyperplasia. Lancet 365: 2125–2136. 15964450 10.1016/S0140-6736(05)66736-0

[3] White PC, Speiser PW (2000) Congenital adrenal hyperplasia due to 21-hydroxylase deficiency. Endocr Rev 21: 245–291. 10857554 10.1210/edrv.21.3.0398

[4] Debono M, Ross RJ, Newell-Price J (2009) Inadequacies of glucocorticoid replacement and improvements by physiological circadian therapy. Eur J Endocrinol 160: 719–729. 19168600 10.1530/EJE-08-0874

[5] Charmandari E, Chrousos GP (2006) Metabolic syndrome manifestations in classic congenital adrenal hyperplasia: do they predispose to atherosclerotic cardiovascular disease and secondary polycystic ovary syndrome? Ann N Y Acad Sci 1083: 37–53. 17148732 10.1196/annals.1367.005

[6] Arlt W, Willis DS, Wild SH, Krone N, Doherty EJ, et al Health status of adults with congenital adrenal hyperplasia: a cohort study of 203 patients. J Clin Endocrinol Metab 95: 5110–5121. 20719839 10.1210/jc.2010-0917 PMC3066446

[7] Mooij CF, Kroese JM, Claahsen-van der Grinten HL, Tack CJ, Hermus AR Unfavourable trends in cardiovascular and metabolic risk in paediatric and adult patients with congenital adrenal hyperplasia? Clin Endocrinol (Oxf) 73: 137–146. 19719762 10.1111/j.1365-2265.2009.03690.x

[8] Reisch N, Arlt W, Krone N Health problems in congenital adrenal hyperplasia due to 21-hydroxylase deficiency. Horm Res Paediatr 76: 73–85. 21597280 10.1159/000327794

[9] Etxabe J, Vazquez JA (1994) Morbidity and mortality in Cushing's disease: an epidemiological approach. Clin Endocrinol (Oxf) 40: 479–484. 8187313 10.1111/j.1365-2265.1994.tb02486.x

[10] Volkl TM, Simm D, Beier C, Dorr HG (2006) Obesity among children and adolescents with classic congenital adrenal hyperplasia due to 21-hydroxylase deficiency. Pediatrics 117: e98–105. 16396852 10.1542/peds.2005-1005

[11] Manenschijn L, van den Akker EL, Lamberts SW, van Rossum EF (2009) Clinical features associated with glucocorticoid receptor polymorphisms. An overview. Ann N Y Acad Sci 1179: 179–198. 19906240 10.1111/j.1749-6632.2009.05013.x

[12] Rosmond R, Chagnon YC, Holm G, Chagnon M, Perusse L, et al (2000) A glucocorticoid receptor gene marker is associated with abdominal obesity, leptin, and dysregulation of the hypothalamic-pituitary-adrenal axis. Obes Res 8: 211–218. 10832763 10.1038/oby.2000.24

[13] Buemann B, Vohl MC, Chagnon M, Chagnon YC, Gagnon J, et al (1997) Abdominal visceral fat is associated with a BclI restriction fragment length polymorphism at the glucocorticoid receptor gene locus. Obes Res 5: 186–192. 9192392 10.1002/j.1550-8528.1997.tb00292.x

[14] Syed AA, Halpin CG, Irving JA, Unwin NC, White M, et al (2008) A common intron 2 polymorphism of the glucocorticoid receptor gene is associated with insulin resistance in men. Clin Endocrinol (Oxf) 68: 879–884. 18194492 10.1111/j.1365-2265.2008.03175.x

[15] Watt GC, Harrap SB, Foy CJ, Holton DW, Edwards HV, et al (1992) Abnormalities of glucocorticoid metabolism and the renin-angiotensin system: a four-corners approach to the identification of genetic determinants of blood pressure. J Hypertens 10: 473–482. 1350793 10.1097/00004872-199205000-00011

[16] van den Akker EL, Koper JW, van Rossum EF, Dekker MJ, Russcher H, et al (2008) Glucocorticoid receptor gene and risk of cardiovascular disease. Arch Intern Med 168: 33–39. 18195193 10.1001/archinternmed.2007.41

[17] Bachega TA, Billerbeck AE, Madureira G, Marcondes JA, Longui CA, et al (1998) Molecular genotyping in Brazilian patients with the classical and nonclassical forms of 21-hydroxylase deficiency. J Clin Endocrinol Metab 83: 4416–4419. 9851787 10.1210/jcem.83.12.5350

[18] Arlt W, Allolio B (2003) Adrenal insufficiency. Lancet 361: 1881–1893. 12788587 10.1016/S0140-6736(03)13492-7

[19] Executive Summary of The Third Report of The National Cholesterol Education Program (NCEP) Expert Panel on Detection, Evaluation, And Treatment of High Blood Cholesterol In Adults (Adult Treatment Panel III). JAMA 285: 2486–2497. 10.1001/jama.285.19.2486 11368702

[20] Karl M, Lamberts SW, Detera-Wadleigh SD, Encio IJ, Stratakis CA, et al (1993) Familial glucocorticoid resistance caused by a splice site deletion in the human glucocorticoid receptor gene. J Clin Endocrinol Metab 76: 683–689. 8445027 10.1210/jcem.76.3.8445027

[21] Gergics P, Patocs A, Majnik J, Balogh K, Szappanos A, et al (2006) Detection of the Bcl I polymorphism of the glucocorticoid receptor gene by single-tube allele-specific polymerase chain reaction. J Steroid Biochem Mol Biol 100: 161–166. 16806906 10.1016/j.jsbmb.2006.04.004

[22] Zhang HJ, Yang J, Zhang MN, Liu CQ, Xu M, et al Metabolic disorders in newly diagnosed young adult female patients with simple virilizing 21-hydroxylase deficiency. Endocrine 38: 260–265. 20978868 10.1007/s12020-010-9382-9

[23] Falhammar H, Filipsson H, Holmdahl G, Janson PO, Nordenskjold A, et al (2007) Metabolic profile and body composition in adult women with congenital adrenal hyperplasia due to 21-hydroxylase deficiency. J Clin Endocrinol Metab 92: 110–116. 17032717 10.1210/jc.2006-1350

[24] Nakazone MA, Pinheiro A, Braile MC, Pinhel MA, de Sousa GF, et al (2007) [Prevalence of metabolic syndrome using NCEP-ATPIII and IDF definitions in Brazilian individuals]. Rev Assoc Med Bras 53: 407–413. 17952349 10.1590/s0104-42302007000500016

[25] Falhammar H, Filipsson Nystrom H, Wedell A, Thoren M Cardiovascular risk, metabolic profile, and body composition in adult males with congenital adrenal hyperplasia due to 21-hydroxylase deficiency. Eur J Endocrinol 164: 285–293. 21098686 10.1530/EJE-10-0877

[26] Mooij CF, Kroese JM, Sweep FC, Hermus AR, Tack CJ Adult patients with congenital adrenal hyperplasia have elevated blood pressure but otherwise a normal cardiovascular risk profile. PLoS One 6: e24204. 10.1371/journal.pone.0024204 PMC3164719 21909422

[27] Arlt W, Krone N (2007) Adult consequences of congenital adrenal hyperplasia. Horm Res 68 Suppl 5: 158–164. 18174737 10.1159/000110615

[28] Tremblay A, Bouchard L, Bouchard C, Despres JP, Drapeau V, et al (2003) Long-term adiposity changes are related to a glucocorticoid receptor polymorphism in young females. J Clin Endocrinol Metab 88: 3141–3145. 12843156 10.1210/jc.2002-021521

[29] Fain JN, Ballou LR, Bahouth SW (2001) Obesity is induced in mice heterozygous for cyclooxygenase-2. Prostaglandins Other Lipid Mediat 65: 199–209. 11444591 10.1016/s0090-6980(01)00136-8

[30] Stenbit AE, Tsao TS, Li J, Burcelin R, Geenen DL, et al (1997) GLUT4 heterozygous knockout mice develop muscle insulin resistance and diabetes. Nat Med 3: 1096–1101. 9334720 10.1038/nm1097-1096

[31] De Iudicibus S, Stocco G, Martelossi S, Drigo I, Norbedo S, et al (2007) Association of BclI polymorphism of the glucocorticoid receptor gene locus with response to glucocorticoids in inflammatory bowel disease. Gut 56: 1319–1320. 10.1136/gut.2006.116160 PMC1954985 17698869

[32] van Rossum EF, Lamberts SW (2004) Polymorphisms in the glucocorticoid receptor gene and their associations with metabolic parameters and body composition. Recent Prog Horm Res 59: 333–357. 14749509 10.1210/rp.59.1.333

